# Starving for nutrients: anorexia during infection with parasites in broilers is affected by diet composition

**DOI:** 10.1016/j.psj.2021.101535

**Published:** 2021-10-13

**Authors:** James Taylor, Panagiotis Sakkas, Ilias Kyriazakis

**Affiliations:** ⁎ Agriculture, School of Natural and Environmental Sciences, Newcastle University, Newcastle on Tyne NE1 7RU, United Kingdom; † CCPA, ZA du Bois de Teillay, 35150 Janzé, France; ‡ Institute for Global Food Security, Queen's University, Belfast BT7 1NN, United Kingdom

**Keywords:** anorexia, broilers, crude protein intake, energy intake, eimeria

## Abstract

In 2 experiments, we investigated whether diet composition plays a role in pathogen-induced anorexia, the voluntary reduction in ADFI during infection in broilers. We hypothesized that either energy or CP dietary content could influence the extent of anorexia in Ross 308 broilers and infection outcomes with *Eimeria maxima*. From d 13 of age, half of the birds were infected, and half were uninfected. ADFI was measured daily, and BW every 3 d until d 29. Oocyst excretion was measured daily from d 17 to 23. The impact of parasitism on the small intestine was assessed on d 19 and 25. In Experiment 1, 336 birds were offered diets progressively diluted with lignocellulose, starting from a diet with 3,105 (kcal ME/kg) and 20% CP. There was a significant interaction between infection and diet on ADFI during the acute stage of infection (d 17 to 21): for control birds diet dilution decreased ADFI and consequently reduced energy and CP intake. For infected birds, diet dilution increased ADFI, leading to the same energy and CP intake across diets. Oocyst excretion and villi length to crypt depth ratio (**VCR**) were constant across infected treatments. In Experiment 2, 432 birds were offered diets with constant ME (3,105 kcal/kg), but different CP contents (24, 20, 26, and 12%). Infection significantly reduced ADFI. Although there was no interaction between infection and diet on ADFI, there was an interaction on CP intake during the acute stage of infection. Infected birds on the 20% CP diet achieved the same CP intake as uninfected birds. There were no differences in the VCR and ADG of the infected birds on 24, 20 and 16% CP treatments, but birds on 12% had the lowest ADG and excreted more oocysts. We suggest that during infection, birds target a nutrient resource intake, which appears to be beneficial for infection outcomes, while at the same time they avoid excess protein intake. We conclude that different mechanisms regulate ADFI in infected and uninfected birds.

## INTRODUCTION

A voluntary reduction in the feed intake of animals and humans is considered an ‘unavoidable’ characteristic of most infections (Adelman and Martin, 2009; Laurenson et al., 2011; Hite and Cressler, 2019). We call this reduction: *pathogen-induced anorexia*. Pathogen-induced anorexia constitutes a “paradox” (Kyriazakis et al., 1998). This is because it occurs at times when host requirements for nutrient resources are increased, rather than decreased (Hite and Cressler, 2019). Pathogens divert resources away from their host and in response to infection hosts initiate resource-demanding processes, such as the immune response and the repair of the damage caused by the pathogen (Sandberg et al., 2007). Because pathogen-induced anorexia is a “hard-wired” behavior across a wide range of infected animals, including humans, it has been hypothesized that it confers evolutionary advantages, usually to the host (Kyriazakis et al., 1998; Rao et al., 2017). Indeed, infected mice that were force fed to increase their intake, succumbed to infection more rapidly and to a greater extent than animals that were allowed to develop the anorexic response (Murray and Murray, 1979).

Knowing how to deal with pathogen-induced anorexia is of interest to several fields, including human and veterinary medicine, and livestock husbandry (Kyriazakis, 2010). This is because knowing what kind of diets to offer to infected hosts during the critical stages of infection will have consequences for the outcomes of the infection and ultimately host survival. An alternative way of viewing pathogen-induced anorexia and reconciling its paradoxical occurrence is to suggest that infected hosts are actually targeting a specific nutrient resource intake through their reduced feed intake. Thus, anorexia may *not* be an unavoidable consequence of infection, but the outcome of an interaction between the host and its diet composition. This is the hypothesis pursued in this paper. We use the term nutrient resource to encompass both energy and nutrients, such as CP, amino acids (**AA**) or minerals. As identified by Kyriazakis (2010), there is a dearth of information about the effect of diet composition on anorexia and, therefore, uncertainty over what is being regulated.

We use the growing broiler chicken and its coccidian, intestinal parasite *Eimeria maxima* as our model system to address our hypothesis in 2 experiments. There are several advantages in using this system: the feed intake of growing broilers changes very rapidly over a short period of time; there is a clear reduction in feed intake during the acute stage of infection, and birds are eventually able to overcome their infection, by which time anorexia is no longer observed (Kipper et al., 2013; Sakkas et al., 2018; Oikeh et al., 2019). In the first experiment, we progressively dilute a nutrient dense diet with an inert material (lignocellulose). The consequences of such a dilution are that while the energy content of the diet declines, the energy to nutrient ratio of the diets remains constant. This enables us to test whether infected hosts attempt to regulate nutrient resources through changes in their feed intake. In the second experiment, we address whether pathogen-induced anorexia is sensitive to either dietary energy or CP supply. We test this by progressively diluting the nutrient dense diet with a protein-free ingredient (starch); while such a dilution maintains the energy content of the diets, it results in diets of different CP contents.

In both experiments we assess the consequences of diet composition on the outcomes of the infection, that is, the extent of the damage caused by the parasite, the number of parasites excreted by the host, and the rate of recovery. Understanding which nutrient resources are targeted by infected hosts and the consequences of this on the outcomes of the infection, will have implications on how hosts should be fed during the critical stages of an infection.

## METHODS

### Experimental Design, Diets, and Animals

#### Experiment 1

A total of 336 male Ross 308 d old broiler chicks were used. At d 7 of age the birds were allocated to the dietary treatments until d 29 of age, when the trial concluded. Four diets appropriate for their stage of growth (grower) were formulated (Table 1): a basal diet (No fiber; NF) and 3 diets, diluted with 5% (Low fiber; **LF**), 10% (Medium fiber; **MF**), and 15% (High fiber; **HF**) Arbocel RC Fine lignocellulose (JRS PHARMA, Rosenberg, Germany). The calculated ME content of the NF diet was 3105 kcal/kg fresh and its analyzed CP content was 19.4%. The energy to nutrient ratios in the diets were assumed to be the same, as Arbocel, a natural lignocellulose produced from fresh spruce trees (*Picea* species), is an inert ingredient, characterized by its high Water Holding Capacity (**WHC**). The expectation was that inclusion of lignocellulose would limit feed intake of broilers in the absence of anorexia due to its “bulkiness” (Nascimento et al., 2020; Taylor et al., 2021). This may not be the case in the presence of pathogen-induced anorexia. All diets were offered as mash to be consistent with the diets produced in Experiment 2. Each dietary treatment was replicated in 12 pens and each pen contained 7 birds at the start of the experiment, which was reduced to 6 birds at d 13 of age.

**Table 1:** Major ingredient (>5% inclusion), calculated and analyzed chemical composition of the common (starter) diet offered from d 0 to d 7 of age, and the experimental diets offered from d 8 to d 28 of age in Experiment 1.

	Lignocellulose inclusion (%)				
Ingredients (%)	Common starter	NF (0)	LF (5)	MF (10)	HF (15)
Ground maize	10.0	10.0	9.50	9.00	8.50
Ground wheat	51.5	53.9	51.2	48.5	45.8
Soybean meal (48% CP)	26.0	23.0	21.9	20.7	19.6
Arbocel	-	-	5.00	10.0	15.0
Full fat Soya	5.00	5.00	4.75	4.50	4.25
Limestone	1.25	1.25	1.19	1.13	1.06
L-Lysine HCL	0.40	0.30	0.29	0.27	0.26
DL-Methionine	0.40	0.35	0.33	0.32	0.30
L-Threonine	0.15	0.15	0.14	0.14	0.13
Soya oil	3.00	3.50	3.33	3.15	2.98
Monocalcium phosphate	1.50	1.25	1.19	1.13	1.06
Salt	0.25	0.25	0.24	0.23	0.21
Sodium bicarbonate	0.15	0.15	0.14	0.14	0.13
Premix	0.40	0.40	0.38	0.36	0.34
Titanium dioxide	-	0.50	0.50	0.50	0.50
Total	100	100	100	100	100
Nutrient and chemical composition (%)^a^					
Metabolizable energy (kcal kg^−1^) (calculated)	3,057	3,105	2,938	2,794	2,627
Gross energy (MJ kg^−1^)	16.9	17.0	17.0	17.0	17.0
N × 6.25 (Crude protein; CP)	21.4	19.4	18.5	17.6	16.9
Crude fibre	2.30	2.87	4.51	7.23	10.7
Ether extract (oil A)	5.55	6.05	5.75	5.45	5.14
Total oil (oil B)	6.52	7.32	6.86	6.51	6.30
Ash	6.40	7.60	5.00	5.20	5.40
Water holding capacity (g/g DM)	-	2.40	2.85	3.17	3.64
T-lysine (calculated)	1.43	1.26	1.20	1.13	1.07
Av-lysine (calculated)	1.34	1.17	1.11	1.06	1.01
Methionine (calculated)	0.70	0.63	0.60	0.57	0.53
Methionine + cysteine (calculated)	1.03	0.94	0.89	0.84	0.80
Threonine (calculated)	0.91	0.85	0.80	0.76	0.72
Tryptophan (calculated)	0.25	0.23	0.22	0.20	0.19
Calcium (calculated)	0.95	0.93	0.88	0.83	0.79
Phosphorus (calculated)	0.69	0.65	0.62	0.59	0.55
Av-Phosphorus (calculated)	0.47	0.42	0.40	0.38	0.36
Salt (calculated)	0.31	0.31	0.29	0.28	0.26
Sodium (calculated)	0.18	0.17	0.17	0.16	0.26

a Analyzed composition unless otherwise stated. The basal, high density diet (NF) was diluted with 5%, 10%, or 15% Arbocel lignocellulose to result in the LF, MF and HF diets, respectively.

#### Experiment 2

A total of 432 male Ross 308 d old broiler chicks were used. At d 7 of age the birds were allocated to the dietary treatments until d 29 of age when the trial concluded. Four grower diets were formulated with different estimated levels of CP (Table 2): 24%, 20%, 16%, and 12%, but the same content of ME (3,105 kcal/kg). This was achieved by exchanging corn gluten meal with corn starch. The diets were formulated to have constant CP to Lysine ratios and AA to Lysine ratios. Calcium, phosphorus, vitamins and minerals were constant in each of the grower diets; the nutrient composition of the 20% CP diet was formulated to be similar to the basal diet used in Experiment 1. In order to achieve the latter, we used cellulose powder (Solka-floc; CFF, Temse, Belgium) as a diluent, at a similar inclusion levels across diets. Experimental diets were offered as mash due to the difficulty of producing pelleted diets with high levels of corn gluten meal and corn starch. Each dietary treatment was replicated in 12 pens and each pen contained 9 birds at the start of the experiment, which was reduced to 7 birds at d13 of age.

**Table 2:** Major ingredient (>5% inclusion), calculated and analyzed chemical composition of the common (starter) diet offered from d 0 to d 7 of age, and the experimental diets offered from d 8 to d 28 of age in Experiment 2.

		Crude protein content (%)			
Ingredients (%)	Common starter	24	20	16	12
Ground maize	10.0	41.1	40.4	39.7	39.1
Ground wheat	51.5	-	-	-	-
Corn starch	-	10.0	16.7	23.3	30.0
Corn gluten meal	-	32.6	26.4	20.2	14.0
Soybean meal (48% CP)	26.0	-	-	-	-
Full fat Soya	5.00	-	-	-	-
Powdered cellulose (Solka-floc)	-	8.43	8.71	8.98	9.26
Limestone	1.25	1.34	1.34	1.35	1.35
L-Lysine HCL	0.40	1.15	0.97	0.78	0.60
DL-Methionine	0.40	0.02	0.03	0.03	0.04
L-Threonine	0.15	0.14	0.13	0.11	0.10
L-Tryptophan	-	0.10	0.08	0.07	0.05
L Arginine	-	0.64	0.54	0.44	0.34
Valine	-	0.00	0.01	0.03	0.04
Iso-leucine	-	0.04	0.05	0.06	0.07
Soya oil	3.00	2.00	2.17	2.33	2.50
Monocalcium phosphate	1.50	1.45	1.50	1.55	1.60
Salt	0.25	0.25	0.25	0.25	0.25
Sodium bicarbonate	0.15	0.35	0.35	0.35	0.35
Premix	0.40	0.40	0.40	0.40	0.40
Titanium dioxide	-	0.50	0.50	0.50	0.50
Total	100	100	100	100	100
Nutrient and chemical composition (%)^a^					
Metabolizable energy (kcal kg-1) (calculated)	3,057	3,105	3,105	3,105	3,105
Gross energy (MJ kg-1)	16.9	17.0	17.0	17.7	18.1
N × 6.25 (Crude protein; CP)	21.4	25.0	21.9	14.8	12.6
Crude fibre	2.90	8.94	9.11	9.28	9.45
Ether extract (oil A)	5.55	4.20	4.27	4.33	4.40
Total oil (oil B)	6.52	5.76	5.81	6.61	7.18
Ash	6.40	4.70	4.20	4.20	4.70
T-lysine (calculated)	1.43	1.41	1.18	0.96	0.73
Av-lysine (calculated)	1.34	1.34	1.13	0.92	0.70
Methionine (calculated)	0.70	0.64	0.54	0.44	0.34
Methionine + cysteine (calculated)	1.03	1.06	0.89	0.73	0.56
Threonine (calculated)	0.91	0.94	0.79	0.65	0.50
Tryptophan (calculated)	0.25	0.21	0.18	0.14	0.11
Calcium (calculated)	0.95	0.89	0.90	0.91	0.92
Phosphorus (calculated)	0.69	0.60	0.58	0.56	0.54
Av-Phosphorus (calculated)	0.47	0.44	0.44	0.44	0.44
Salt (calculated)	0.31	0.30	0.31	0.31	0.32
Sodium (calculated)	0.18	0.21	0.21	0.21	0.21

a Analyzed composition unless otherwise stated. For the basal, high density diet (24%) corn starch was replaced with corn gluten meal, to result in iso-energetic diets with crude protein contents of 20%, 16%, and 12%.

#### Common Protocol

All procedures were conducted under the UK Animals (Scientific Procedures) Act 1986 and EU Directive 2010/63/EU for animal experiments and carried out under UK Home Office authorization (P441ADF04). Furthermore, all procedures were approved by the Animal Welfare and Ethical Review Body (AWERB) of Newcastle University. All chicks were obtained from a commercial hatchery. They were housed in a thermostatically controlled building in 48 pens, each with an area of 0.85m^2^. All birds were wing tagged upon arrival. Pens were equipped with feeders and drinkers, with wood shavings used as litter at a depth of 5 cm. The birds had free and continuous access to feed and water throughout the trial. The pen temperature was set to 34°C at arrival and was gradually reduced to 20°C by d 25 of age. The lighting schedule was 23 h Light (L):1 h Darkness (D) for the first 7 d and was amended to 18L:6D for the course of the trial, while light intensity at pen level ranged from 80 to 100 lux.

All birds were fed the same starter diet until d 7 of age (Tables 1 and 2). To reduce the risk of refusals and starve outs the birds were offered a mixture of one-part common starter and one-part experimental diet between d 8 and d 10 of age, and the experimental diet only from d 11. Birds were weighed individually at arrival (d0), at d7 and then individually every 3 d until the end of the trial. Pen feed intake was measured between d 0 and d 7 and then daily until the conclusion of the experiment.

On d 13 of age (d 0 postinfection; **pi**), birds were orally inoculated with either a single dose of 0.5 mL of H_2_O (controls), or 7,000 sporulated *Eimeria maxima* oocysts of the Weybridge laboratory reference strain suspended in H_2_O (infected), so that within a dietary treatment half of the birds were control and the other half were infected. A previously developed biosecurity protocol (Sakkas et al., 2018) was carefully followed to ensure that there was no cross-contamination between infected and uninfected (control) birds. In order to ensure this, measurements on the control birds were taken first, and PPE and other equipment were changed before taking the measurements on the infected birds.

As the 2 experiments were not contemporaneous, the *Eimeria* batch used in the second experiment was obtained at a later time. In both experiments the batches were approximately 3 months of age. *E. maxima* was chosen as it enables us to address the objectives of the experiments. The pathogen induces replicable anorexia on standard diets; such anorexia is relatively short in duration and is eventually overcome (Kipper et al., 2013; Sakkas et al., 2018; Oikeh et al., 2019).

#### Sampling

On d 6 and 12 pi, one bird from each pen with a BW close to the pen average was culled by intravenous lethal injection with sodium pentobarbital (Euthatal, Merial Harlow, United Kingdom); the sampling times corresponded to the acute and recovery stages of the infection. During necropsy, the full gastrointestinal tract (**GIT**) was removed and separated into individual segments of interest. After measuring the length of the duodenum, jejunum and ileum, a small section (1–2 cm) of duodenum and jejunum was collected and stored in 10% neutral buffered formalin for morphometric analysis. The duodenal sample was collected from the middle of the duodenal loop; and the jejunal sample from halfway between the point of entry of the bile ducts, and Meckel's diverticulum.

### Sample Analysis

#### Diet Analysis

Samples from all diets were analyzed for gross energy, CP, crude fiber, ash, ether extract and total oil. All analyses were performed at a UKAS accredited commercial laboratory to the internationally recognized standard for competence (Sciantec Analytical Services, Cawood, UK).

#### Oocyst Production

Excreta were collected from d 4 to 10 pi by placing polyethylene sheets on top of the wood shavings of each pen for 90 min, from both infected and control pens. Upon sheet removal, excreta were pooled per pen into screw cap pots and stored at 4°C, pending analysis. The modified McMaster technique was used to estimate excretion of daily oocysts per gram (Kaufmann, 2013).

The feces were thoroughly mixed before a 3 g sample was removed and the remaining fecal material was freeze-dried to estimate DM content. The sample was mixed with 42 mL of water and passed through a sieve. The solution was then transferred to a glass test tube and was centrifuged at 1500 RPM for 2 min at room temperature. The supernatant was carefully siphoned off and the pellet was vortexed until it was fragmented. Then 10 mL of saturated NaCl were added and the solution was thoroughly mixed. A sample of the solution was taken from the center of the tube before being carefully transferred to the McMaster counting slide. Slides were left undisturbed for 10 min to allow the oocysts to rise to the top of the slide, before being read at 10 × magnification. The sum of the oocyst count of each chamber was calculated and multiplied by 50 to give oocysts per gram.

#### Histology

Intestinal segments fixed in formalin from the duodenum and jejunum were subjected to a series of graded ethanol baths followed by xylene in a Shandon Excelsior Es Tissue Processor (Thermo Fisher Scientific Inc., Waltham, MA) in order to dehydrate the tissue. The samples were then embedded in paraffin wax, sectioned at 4 µm and stained with hematoxylin/eosin. After staining, the histological sections were scanned using the Leica SCN400 slide scanner system (Leica Microsystems, Buffalo Grove, IL) and images were captured using the Leica SCN400 image viewer software. Morphometric features of the intestinal structure were observed at 10 × magnification. Villus height and crypt depth measurements were ascertained using Aperio ScanScope CS (Aperio Technologies, Vista, CA). The vertical distance from the villus tip to the villus-crypt junction of 10 villi was used to determine villus height, while crypt depth was the vertical distance from the villus-crypt junction to the lower limit of the crypt of 10 corresponding crypts. Villi length to crypt depth ratio (**VCR**) was calculated as the usual measure of cell proliferation, which reflects the extent of intestinal damage (Kettunen et al., 2001).

### Calculations and Statistics

Pen (*n* = 6) was considered the experimental unit for all data and all statistical analysis was performed using the nlme package in R (Team, 2013) using lm and anova functions from the nlme package (Pinheiro et al., 2013). For all statistical procedures, the normality of the residuals was assessed with qq-plots and the Shapiro-Wilk test. When significant differences were detected, treatment means were separated and compared by the Tukey's multiple comparison test. Significance was determined at *P < 0.05*. Data are presented as model-predicted least square means with the SEM.

Pen average daily CP intake (g/d) and average daily energy intake (energy intake, kcal/d) were calculated by multiplying ADFI (g/d) by the CP level and the energy content of the diet respectively.

In the first instance, a repeated measures mixed model was implemented to analyze daily feed intake, as recorded. The model included diet, infection, and day as fixed factors, 2-way interactions between diet and infection, diet, and day, infection and day and the 3-way interaction between diet, infection, and day. Covariance structures were chosen based on the lowest value for the Akaike and Bayesian information criteria. Further analysis of variance was carried out on time points when the 3-way interaction between diet, infection, and day was significant. This allowed us to identify 3 stages in relation to the development of daily feed intake: 1) prepatent; 2) acute stage of infection, and 3) recovery.

ADFI, nutrient resource intake (i.e., average daily energy intake and daily CP intake) and ADG were calculated for each of these 3 stages of the infection. Each of these variables for each phase was analyzed with GLM models with diet and infection as fixed factors and their interactions. Diet and infection affected the growth trajectories of the bird during the course of the experiment and as a consequence birds were of different BW at the start of each stage of infection. For this reason BW at the start of each stage was used as a covariate since there is a linear relationship between performance variables and BW (Allison, 1995; Packard and Boardman, 1999). Where no significant interactions were observed between infection and diet, performance variables were assessed for linear and quadratic relationships using diet as the explanatory variable.

Histological measurements obtained from one bird per pen were obtained at d 6 and d12pi, were expressed relative to the BW of the bird prior to sampling (µm/kg BW) to account for *a priori* differences in performance (Sakkas et al., 2018). Relative lengths of the GIT segments, relative villi lengths, relative crypt depths and the villi lengths to crypt depth ratios were analyzed with GLM models with diet and infection as fixed factors and their interactions. The measured DM of daily excreta was used to express oocyst excretion per g DM, to account for differences in the consistency of the feces produced. Oocyst excretion was analyzed by a repeated measures mixed model with diet, time and their interactions as fixed factors. Infection status was not included in this repeated measures model since no oocysts were observed in the control pens of either experiments. Oocyst per g DM data needed to be transformed by the natural logarithm prior to statistical analysis, as the residuals were not normally distributed.

## RESULTS

### Experiment 1

#### Feed and Nutrient Resource Intake

**Figure 1:**
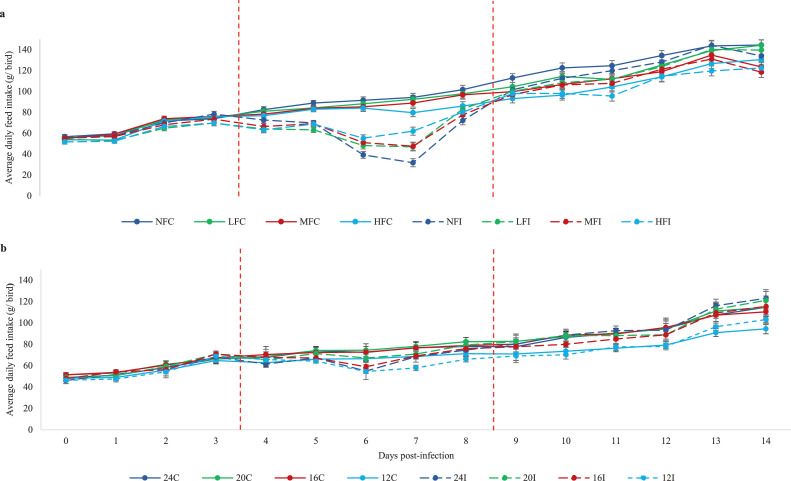
Daily feed intake of broilers infected with *Eimeria maxima* oocysts (a) Experiment 1 - Birds were offered diets with the same ME:CP ratio, diluted with 0% (NF), 5% (LF), 10% (MF), or 15% (HF) Arbocel lignocellulose; (b) Experiment 2 - Birds were offered isoenergetic diets with 24%, 20%, 16%, or 12% CP. The intakes (g/bird) of both the infected (I) and the corresponding uninfected control (C) birds are shown. Dotted vertical lines indicate separate stages of the infection defined by a repeated measures ANOVA: prepatent (d0-3pi), acute (d4-8pi), and recovery (d9-14pi).

ADFI (g/d) was not statistically affected by the infection until d 4 and recovered from d 9 pi onward (Figure 1A). Therefore, d 0 to 3, 4 to 8, and 9 to 14 pi were considered to represent the prepatent, acute and recovery stages of infection respectively.

The ADFI of the infected birds was significantly reduced during the perpatent and acute stages of infection (*P* < 0.001), compared to the uninfected birds, and the daily energy and CP intakes of the infected birds were significantly reduced during all stages of infection (Table 3, *P* < 0.001). Diet significantly affected ADFI during the prepatent (*P =* 0.011) and recovery stages (*P* < 0.001); while ADFI increased significantly on the diets with the highest Arbocel dilution (MF and HF) during the prepatent stage, the reverse was the case during the recovery stage (i.e., ADFI was higher on NF and LF). Diet dilution significantly reduced daily energy and CP intakes during all stages of the infection *(P* < 0.001*).* There was a significant interaction between infection and diet during the acute stage of infection on ADFI (*P* < 0.001). While ADFI of the uninfected birds was not significantly different between the 4 diets, ADFI of the infected birds increased as diet was progressively diluted; as a consequence, the daily ADFI on NF and HF diets was 52.8 vs. 70.3 g/d respectively. Similarly, there was a strong tendency for an interaction between infection and diet on daily energy and CP intake during the acute stage of infection (*P* = 0.051 in both cases). While there were no significant differences in the resource intakes of the infected birds, the resource intakes of the uninfected birds were affected by diet composition; both daily energy and CP intakes decreased as diet was progressively diluted with Arbocel.

**Table 3:** ADFI, energy and CP intakes of the infected and the corresponding uninfected control birds during infection with *Eimeria maxima* oocysts in Experiment 1.

		ADFI (g/d)			Energy intake (kcal/d)			CP intake (g/d)			ADG (g/d)		
Treatment		Prepatent	Acute	Recovery	Prepatent	Acute	Recovery	Prepatent	Acute	Recovery	Prepatent	Acute	Recovery
Diet													
	NF	62.4^b^	69.5	120^ab^	201^a^	218^a^	304^a^	12.9^a^	14.0^a^	19.5^a^	37.8^a^	60.2^ab^	64.7^a^
	LF	62.9^b^	74.8	122^a^	191^ab^	211^ab^	295^ab^	12.2^ab^	13.5^ab^	19.0^ab^	34.8^ab^	62.9^a^	61.5^ab^
	MF	64.7^a^	74.8	118^b^	185^ab^	204^b^	279^bc^	11.9^b^	13.1^b^	17.9^bc^	33.7^b^	54.9^b^	57.3^bc^
	HF	65.2^a^	77.8	118^b^	177^b^	202^b^	267^c^	11.4^b^	13.0^b^	17.1^c^	32.6^b^	54.7^b^	52.6^c^
	SEM	0.87	1.67	2.2	2.8	3.5	5.3	0.18	0.22	0.34	0.874	1.81	1.8
Infection													
	Control	64.9	86.3	121	194	233	293	12.5	15.0	18.8	35.3	68.6	62.2
	Infected	62.7	62.1	118	182	185	279	11.7	11.9	17.9	34.1	47.8	55.8
	SEM	0.56	1.04	1.4	1.8	2.2	3.5	0.12	0.14	0.23	0.56	1.13	1.20
Diet × Infection													
Uninfected	NF	62.7	86.3^a^	122	205	249	321	13.2	16.0	20.6	37.6	79.7^a^	71.5
	LF	63.9	86.9^a^	125	197	236	299	12.7	15.1	19.2	35.9	70.1^b^	63.3
	MF	66.3	86.9^a^	119	192	226	285	12.3	14.5	18.3	34.5	65.6^bc^	60.0
	HF	66.7	85.3^a^	119	183	221	268	11.8	14.2	17.2	33.2	58.9^cd^	53.9
Infected	NF	62.1	52.8^d^	118	197	187	286	12.7	12.0	18.4	38.0	40.6^e^	58.0
	LF	61.8	62.8^c^	119	184	186	291	11.8	11.9	18.7	33.6	55.8^d^	59.6
	MF	63.1	62.7^c^	116	178	182	273	11.4	11.7	17.5	32.9	44.2^e^	54.5
	HF	63.7	70.3^b^	118	170	183	265	11.0	11.8	17.1	32.0	50.4^de^	51.2
	SEM	1.17	2.18	2.8	3.8	4.5	6.8	0.24	0.29	0.44	1.17	2.28	2.34

Source		*Probabilities*											

Diet		0.011	0.907	**<**0.001	**<**0.001	**<**0.001	**<**0.001	**<**0.001	**<**0.001	**<**0.001	**<**0.001	**<**0.001	**<**0.001
Infection		0.001	**<**0.001	0.058	**<**0.001	**<**0.001	**<**0.001	**<**0.001	**<**0.001	**<**0.001	0.053	**<**0.001	**<**0.001
Diet × Infection		0.649	0.001	0.746	0.828	0.051	0.102	0.828	0.051	0.102	0.688	**<**0.001	0.086

a-e Means within a column that do not share a common superscript are significantly different (*P* < 0.05). LS means with SEM of ADFI (g/d), energy intake (kcal/d), CP intake (g/d), and ADG (g/d) with BW at the start of the stage of infection as a covariate. Data are presented over the prepatent (d 0–3 pi), acute (d 4–8 pi), and recovery stages (d 9–14pi). Birds were offered diets diluted with 0% (NF), 5% (LF), 10% (MF), or 15% (HF) Arbocel lignocellulose.

#### Infection Outcomes

**Figure 2:**
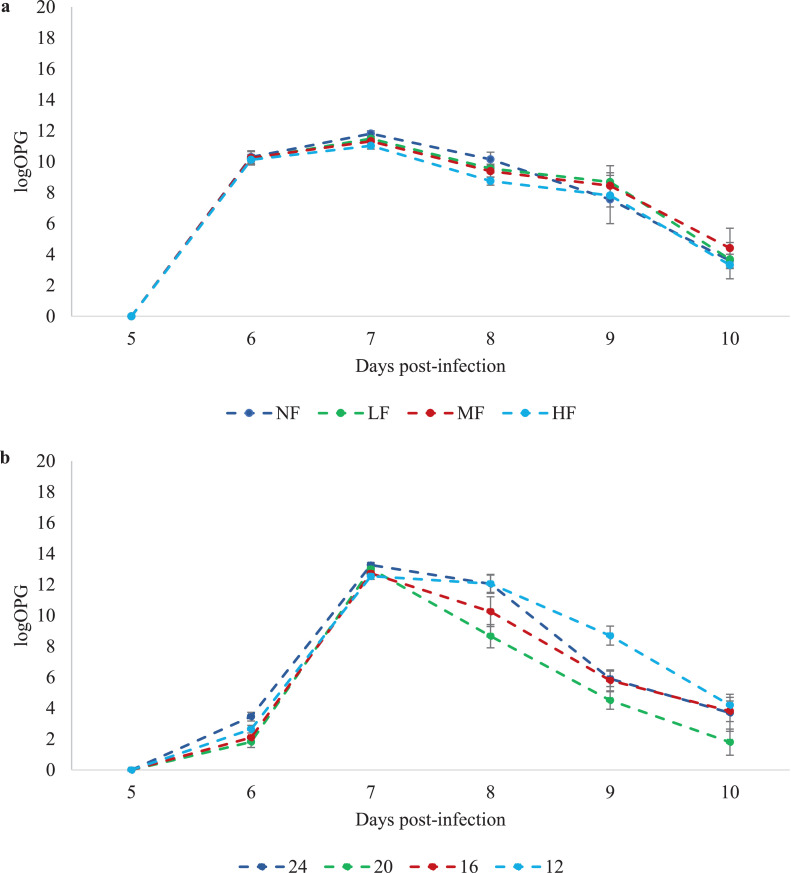
Number of oocysts per gram of fecal DM (OPG) on a logarithmic scale, for birds infected with 7000 *Eimeria maxima* oocysts offered (A) Experiment 1 - Birds were offered diets with the same ME:CP ratio, diluted with 0% (NF), 5% (LF), 10% (MF), or 15% (HF) Arbocel lignocellulose; diet NF contained 3105 kcal/kg ME and 19.4% CP, or (B) Experiment 2 - Birds were offered isoenergetic diets (3105 kcal/kg ME) with 24%, 20%, 16%, or 12% CP. No oocysts were detected in the feces of the corresponding control birds.

There were no mortalities from d 11 of age until the conclusion of the experiment. The effect of diet composition on resistance and resilience to *Eimeria* infection were assessed by measuring oocyst production, ADG and VCR. There was no evidence of infection among the uninfected control birds as there were no oocysts present in the feces from their pens, confirming the absence of contamination between infected and control pens. Excreta were collected from d4pi and infected birds began to excrete oocysts from d6pi and were virtually zero by d 10 pi (Figure 2A). There was no effect of diet or an interaction between diet and time on oocyst excretion/g DM (*P* > 0.05); the across time back transformed means and confidence intervals for oocysts/g DM were 2,392 (1,164–4,915), 2,515 (1,212–5,167), 2,592 (1,249–5,324), and 3,533 (1,703–7,332) for dietary treatments NF, LF, MF and HF, respectively.

BW throughout the infection are given in Figure S1. Infection significantly reduced ADG during the acute and recovery stages of the infection (Table 3, *P* < 0.001). Diet significantly affected ADG during all stages of infection (*P* < 0.001), with MF and HF birds having the lowest ADG compared to the other 2 dietary treatments. There was a significant interaction between infection and diet (*P* < 0.001) during the acute stage of the infection only, due to the fact that as dilution increased ADG decreased linearly of the uninfected birds, whereas there was no significant difference in the performance of the NF, MF, and HF infected birds.

Sections of the duodenum and jejunum were collected from one bird per pen at d 6 and d12pi. The effects on VCR, a measure of intestinal damage, were similar for the jejunum and duodenum. Infection significantly affected crypt depth (*P* < 0.001), but not villi length, in the duodenum and jejunum (µm/kg BW) at d6pi (Table 4); infected birds had deeper crypts in comparison to uninfected control birds. Similarly, at d12pi (Table 4) duodenal and jejunal crypts were deeper in infected birds than controls (*P* < 0.001). Infection significantly increased villi length in the jejunum at d12pi (*P* = 0.012). VCR was significantly reduced in infected birds in the duodenum and jejunum at both d6pi (*P* < 0.001) and d12pi (*P* = 0.002*, P* = 0.003*,* for the duodenum and jejunum respectively). Jejunal villi length was significantly affected by diet on both d6pi (*P* = 0.007) and d12pi (*P* = 0.009); birds fed diluted diets had longer villi than NF fed birds.

**Table 4:** Histology measurements from Experiment 1 of the infected and the corresponding uninfected control birds at the acute (d6pi) and recovery (d12pi) stages of infection with *Eimeria maxima*.

		d6pi						d12pi					
		Duodenum			Jejunum			Duodenum			Jejunum		
Treatment		VCR	Villi length (µm/kg BW)	Crypt depth (µm/kg BW)	VCR	Villi length (µm/kg BW)	Crypt depth (µm/kg BW)	VCR	Villi length (µm/kg BW)	Crypt depth (µm/kg BW)	VCR	Villi length (µm/kg BW)	Crypt depth (µm/kg BW)
Diet													
	NF	9.51	1,918	239	5.43	1,029^b^	223	13.0	1,535	126	6.45	771^b^	127^ab^
	LF	8.33	2,290	302	5.21	1,318^ab^	307	12.9	1,571	125	8.24	972^ab^	123^b^
	MF	9.21	2,118	272	5.79	1,417^a^	270	11.4	1,701	158	7.27	1,003^a^	152^ab^
	HF	9.23	2,171	285	6.12	1,344^a^	264	12.1	1,718	144	7.09	1,066^a^	158^a^
	SEM	0.663	122	17.6	0.431	79.7	23.6	0.93	102	8.9	0.713	58	10.5
Infection													
	Control	12.2	2,181	184	7.39	1,234	172	13.8	1,573	119	8.33	879	107
	Infected	5.99	2,068	364	3.88	1,320	360	10.9	1,689	158	6.19	1,027	173
	SEM	0.468	86.4	12.5	0.305	56.3	16.7	0.63	68.7	6.0	0.489	41.3	7.2
Diet × Infection													
Uninfected	NF	13.3	2,078	159	7.04	1,002	155	16.2	1,547	96	7.47	635	83.1
	LF	10.2	2,085	208	6.98	1,238	181	13.7	1,434	110	8.91	921	105
	MF	11.9	2,284	195	7.22	1,359	193	12.8	1,710	139	8.53	923	117
	HF	13.1	2,277	174	8.32	1,338	160	12.4	1,602	131	8.43	1,035	123
Infected	NF	5.71	1,758	318	3.81	1,056	292	9.81	1,524	157	5.44	906	170
	LF	6.45	2,496	396	3.44	1,399	434	12.1	1,708	141	7.57	1,023	140
	MF	6.50	1,952	349	4.35	1,475	348	9.92	1,692	176	6.02	1,082	187
	HF	5.31	2,066	395	3.92	1,351	368	11.8	1,833	157	5.76	1,098	193
	SEM	0.937	172	24.9	0.61	113	33.3	1.31	144	12.6	1.104	89.8	16.3

Source		*Probabilities*											

Diet		0.627	0.196	0.090	0.472	0.007	0.111	0.628	0.408	0.053	0.33	0.009	0.041
Infection		**<**0.001	0.359	**<**0.001	**<**0.001	0.287	**<**0.001	0.002	0.291	**<**0.001	0.003	0.012	**<**0.001
Diet × Infection		0.116	0.114	0.530	0.637	0.917	0.311	0.131	0.593	0.500	0.900	0.608	0.323

a-b Means within a column that do not share a common superscript are significantly different (*P* < 0.05). Villi length (µm/kg BW at the point of euthanasia), crypt depth (µm/kg BW), and villi length to crypt depth ratio (VCR) of the duodenum and jejunum. Birds were offered diets diluted with 0% (NF), 5% (LF), 10% (MF), or 15% (HF) Arbocel lignocellulose.

Measurements of the small intestine segments were obtained from one bird per pen at d 6 and d12pi (Table S1). Infected birds had higher relative lengths of all intestinal segments compared with control birds at d6 and d12pi (*P* < 0.001 for all variables with the exception of *P* = 0.003 for the duodenum on d6pi and *P* = 0.001 for the jejunum on d12pi). Diet significantly affected relative intestinal lengths of all segments on d6pi: as diet was progressively diluted with Arbocel, relative lengths of all segments progressively increased. This diet effect was significant for the duodenum (*P* = 0.019), jejunum (*P* < 0.001), ileum (*P* < 0.001) on d6pi and for the duodenum segment on d12pi (*P* = 0.003). Moreover, there was an interaction between diet and infection in the relative lengths of all intestinal sections on d6pi (*P* = 0.028 for the duodenum, *P* < 0.001 for the jejunum, and *P* = 0.002 for the ileum). Infected birds increased their relative segment lengths as diet was progressively diluted, whereas in the absence of infection there were no differences between the dietary treatments.

### Experiment 2

#### Feed and Nutrient Resource Intake

ADFI (g/d) was not statistically affected by the infection until d 4 and recovered from d 9 pi onward (Figure 1B). Therefore, d 0 to 3, 4 to 8, and 9 to 14 pi were considered to represent the prepatent, acute, and recovery stages of infection respectively.

ADFI (g/d), daily energy (kcal/d), and CP (g/d) intakes of the infected birds were significantly lower (*P* < 0.001) than the intakes of the uninfected birds during the acute stage of infection (Table 5; ADFI, g/d). Diet significantly affected ADFI, daily energy and CP intakes during all stages of infection (*P* < 0.001 for all variables with the exception of *P* = 0.002 for energy intake during the prepatent stage). ADFI and energy intake increased, and CP intake decreased as the CP of the diets decreased, with the effects being linear during the prepatent stage of infection for all variables (*P* = 0.035 for all variables with the exception of *P* < 0.001 for CP intake) and on CP intake only during the recovery stage of infection (*P* < 0.001). There were also quadratic effects of diet on CP intake during the prepatent and recovery stages of infection (*P* < 0.001), whereas the quadratic relationship was evident for ADFI and energy intake during all stages of infection (*P* < 0.001 with the exception of *P* = 0.009 and *P* = 0.004 for ADFI and energy intake respectively). There was no interaction between infection and diet on ADFI and energy intake during any of the stages of the infection. However, there was an interaction (*P* < 0.001) between infection and diet on the CP intake during the acute stage of the infection. Control uninfected birds significantly reduced their intake as the CP of the diet was reduced; this was not the case for the infected birds, as the CP intakes between infected birds on the 24 and 20% dets was similar. Infected birds showed the greatest reduction in their CP intake on the 24% diet compared to their uninfected controls; infected birds on the 20% CP diet showed no significant difference in CP intake compared to their uninfected counterparts.

**Table 5:** ADFI, energy and CP intakes of the infected and the corresponding uninfected control birds during infection with *Eimeria maxima* oocysts in Experiment 2.

		ADFI (g/d)			Energy intake (kcal/d)			CP intake (g/d)			ADG (g/d)		
Treatment		Prepatent	Acute	Recovery	Prepatent	Acute	Recovery	Prepatent	Acute	Recovery	Prepatent	Acute	Recovery
Diet													
	24	54.7^b^	63.8^b^	85.0^b^	166^b^	198^b^	264^c^	13.5^a^	15.9^a^	21.6^a^	27.7^a^	38.8^ab^	46.4^a^
	20	57.5^ab^	70.8^a^	89.9^ab^	175^ab^	218^a^	279^b^	11.8^b^	14.4^b^	18.8^b^	25.8^a^	40.1^a^	47.8^a^
	16	59.7^a^	73.2^a^	97.2^a^	182^a^	225^a^	302^a^	9.71^c^	11.7^c^	15.4^c^	23.3^b^	39.9^a^	43.5^b^
	12	58.4^ab^	73.0^a^	97.7^a^	179^a^	223^a^	303^a^	7.22^d^	8.88^d^	11.8^d^	19.6^c^	36.7^b^	38.0^c^
	SEM	1.39	1.21	2.58	2.6	3.0	3.1	0.215	0.361	0.43	0.98	0.88	0.99
Infection													
	Control	58.9	74.3	92.2	176	227	286	10.6	13.5	16.8	23.8	47.9	43.2
	Infected	56.2	66.1	92.7	175	205	288	10.5	12.0	17.0	24.4	39.9	34.6
	SEM	0.46	0.62	0.62	1.5	1.9	1.9	0.07	0.09	0.10	0.32	0.45	0.45
Diet × Infection													
Uninfected	24	56.0	69.0	83.7	167	211	260	13.7	17.2^a^	21.4	27.8	42.8	50.2
	20	58.9	74.2	89.9	176	226	279	11.9	15.0^b^	18.7	25.9	44.8	50.9
	16	61.5	77.0	97.6	183	236	303	9.82	12.3^c^	15.5	23.0	44.2	48.2
	12	59.4	76.9	97.5	179	233	303	7.20	9.43^e^	11.8	18.6	40.9	42.4
													
Infected	24	53.3	58.7	86.2	166	184	268	13.3	14.6^b^	21.9	27.7	34.8	42.7
	20	56.0	67.5	89.9	174	210	279	11.8	13.9^b^	18.8	25.7	35.4	44.6
	16	58.0	69.4	96.8	180	215	300	9.61	11.1^d^	15.3	23.6	35.7	38.8
	12	57.4	69.0	97.9	178	213	304	7.24	8.33^f^	11.8	20.7	32.6	33.6
	SEM	1.55	2.61	2.66	4.2	6.3	7.1	0.238	0.409	0.462	1.09	2.02	1.90

Source		*Probabilities*											

Diet		**<**0.001	**<**0.001	**<**0.001	0.002	**<**0.001	**<**0.001	**<**0.001	**<**0.001	**<**0.001	**<**0.001	**<**0.001	**<**0.001
Infection		**<**0.001	**<**0.001	0.423	0.491	**<**0.001	0.423	0.264	**<**0.001	0.289	0.187	**<**0.001	**<**0.001
Diet × Infection		0.884	0.512	0.592	0.978	0.472	0.591	0.638	**<**0.001	0.327	0.279	0.322	0.878

a-f Means within a column that do not share a common superscript are significantly different (*P* < 0.05). LS means with SEM of ADFI (g/d), energy intake (kcal/d), CP intake (g/d) and ADG (g/d) with BW at the start of the stage of infection as a covariate. Data are presented over the prepatent (d0-3pi), acute (d4-8pi), and recovery stages (d9-14pi). Birds were offered diets with 24%, 20%, 16%, or 12% CP.

#### Infection Outcomes

There were no mortalities from d 11 of age until the conclusion of the experiment. The effect of diet composition on resistance and resilience to *Eimeria* infection were assessed by measuring oocyst production (resistance), ADG and VCR (resilience). There was no evidence of infection among the uninfected control birds as there were no oocysts present in the feces from their pens, confirming the absence of contamination between infected and control pens. Excreta were collected from d 4 pi and infected birds began excreting oocysts from d6pi and were virtually zero by d 9 pi (Figure 2B). There was a statistical effect of diet (*P* < 0.001) and an interaction between diet and time (*P < 0.05*) on oocyst excretion/g DM. The across time back-transformed means and confidence intervals for oocyst excretion/g DM were 1,380 (620–3,041), 5,014 (2,208–11,384), 6,374 (2,893–14,186), 9,997 (4,403–22,697) for the 24%, 20%, 16% and 12% dietary treatments, respectively. The interaction between day and diet was due to the 12% CP treatment excreting significantly more oocysts/g DM at d 9 pi, compared to the 20% CP treatment (*P* < 0.05).

BW throughout the infection are given in Figure S2. Infection significantly reduced the ADG during the acute and recovery stages of the infection (Table 5, *P* < 0.001). Diet significantly affected the ADG (*P* < 0.001) during all stages of the infection, with birds on the 12% CP diet having the lowest ADG compared to the other dietary treatments. The effect of diet on ADG was linear during the prepatent stage of infection only (*P* = 0.011), whereas there was a quadratic effect during all stages of infection (*P* < 0.001). The ADG of the birds on the 24% and 20% CP did not differ during any of the stages of the infection (*P* > 0.05). There was no interaction between infection and diet on the adjusted average daily gain during any of the stages of infection (*P* > 0.05).

Sections of the duodenum and jejunum were collected from one bird per pen at d 6 and d12pi. The effects on VCR, a measure of intestinal damage, were similar for the jejunum and duodenum. Infection caused significant increases in crypt depth at both time points, in both the duodenum and jejunum (*P* < 0.001). At d6pi (Table 6), infection caused significant reductions in villi length (µm/kg BW) and VCR in the duodenum and jejunum (*P* < 0.001 for all variables with the exception of *P* = 0.002 for jejunal villi length). Reducing dietary CP caused significant increases in VCR (*P* = 0.048) and villi length (*P* < 0.001) only in the duodenum at d6pi. At d12pi (Table 6), duodenal and jejunal VCR remained significantly lower in infected birds (*P* < 0.001); and infected birds had deeper crypts in comparison to the uninfected control birds in both the duodenum and jejunum (*P* < 0.001). At d12pi, reducing dietary CP increased villi length in the duodenum and jejunum (*P* < 0.001); and increased crypt depth (µm/kg BW) in the jejunum (*P* < 0.001). There was an interaction at d12pi, as infected birds on the 12% CP diet had larger crypt depths than their uninfected counterparts (*P* = 0.043).

**Table 6:** Histology measurements from Experiment 2 of the infected and the corresponding uninfected control birds at the acute (d6pi) and recovery (d12pi) stages of infection with *Eimeria maxima*.

		d6pi						d12pi					
		Duodenum			Jejunum			Duodenum			Jejunum		
Treatment		VCR	Villi length (µm/kg BW)	Crypt depth (µm/kg BW)	VCR	Villi length (µm/kg BW)	Crypt depth (µm/kg BW)	VCR	Villi length (µm/kg BW)	Crypt depth (µm/kg BW)	VCR	Villi length (µm/kg BW)	Crypt depth (µm/kg BW)
Diet													
	24	7.49^b^	2,186^b^	352	5.88	1,531	300	10.2	1,851^c^	190	7.15	1,133^b^	162^b^
	20	8.76^ab^	2,361^b^	323	5.51	1,482	298	10.2	1,915^bc^	200	6.68	1,113^b^	176^b^
	16	8.71^ab^	2,547^b^	360	5.52	1,504	323	10.8	2,107^b^	217	6.77	1,216^b^	187^ab^
	12	9.68^a^	2,891^a^	361	5.51	1,676	362	12.1	2,523^a^	226	7.51	1,499^a^	217^a^
	SEM	0.530	95.7	26.3	0.42	90.6	18.5	0.696	59.9	10.1	0.409	41.0	10.4
Infection													
	Control	12.3	2,786	232	7.81	1,699	225	13.1	2,094	164	8.04	1,271	162
	Infected	5.01	2,207	467	3.41	1,397	417	8.51	2,104	252	6.01	1,210	209
	SEM	0.375	67.7	18.6	0.297	64.1	13.1	0.492	42.4	7.15	0.289	29.0	7.31
Diet × Infection													
Uninfected	24	10.8	2,545	244	8.16	1,687	214	11.7	1,811	158	7.41	1,163	162^b^
	20	12.3	2,569	212	7.24	1,480	206	12.5	1,948	159	7.84	1,093	143^b^
	16	12.4	2,789	230	7.87	1,697	227	13.7	2,091	159	7.68	1,260	167^b^
	12	13.7	3,240	241	7.97	1,931	251	14.6	2,525	181	9.25	1,567	176^b^
													
Infected	24	4.15	1,828	461	3.61	1,375	386	8.70	1,891	221	6.89	1,103	163^b^
	20	5.20	2,152	435	3.78	1,483	391	7.85	1,882	241	5.52	1,134	208^ab^
	16	5.01	2,305	491	3.17	1,311	418	7.85	2,122	275	5.86	1,172	208^ab^
	12	5.70	2,542	481	3.06	1,421	474	9.64	2,520	271	5.77	1,432	259^a^
	SEM	0.751	135	37.2	0.595	128	26.2	0.984	84.7	14.3	0.578	57.9	14.6

Source		*Probabilities*											

Diet		0.048	<0.001	0.715	0.900	0.435	0.067	0.185	**<**0.001	0.066	0.462	**<**0.001	0.004
Infection		**<**0.001	**<**0.001	**<**0.001	**<**0.001	0.002	**<**0.001	**<**0.001	0.870	**<**0.001	**<**0.001	0.148	**<**0.001
Diet × Infection		0.86	0.606	0.937	0.624	0.241	0.796	0.549	0.850	0.318	0.097	0.488	0.043

a-c Means within a column that do not share a common superscript are significantly different (*P* < 0.05). Villi length (µm/kg BW at the point of euthanasia), crypt depth (µm/kg BW), and villi length to crypt depth ratio (VCR) of the duodenum and jejunum. Birds were offered diets with 24%, 20%, 16%, or 12% CP.

Measurements of the small intestine were obtained from one bird per pen at d 6 and d12pi (Table S2). Infected birds showed greater development of intestinal segments than control birds at d6 and d12pi (*P* < 0.05). Reducing CP content caused an increase in the relative lengths of the duodena, jejuna, and ilea (*P* < 0.05). Birds offered the 12% CP diet yielded significantly larger relative lengths than all other dietary treatments (*P* < 0.05). No interaction between diet and infection was observed at either time point in any of the measurements (*P* > 0.05).

## DISCUSSION

Kyriazakis (2010, 2014) has called for research on the relationship between diet composition and regulation of feed intake in animals infected with macro- and microparasites. Understanding of this relationship will provide much needed information about how hosts need to be dealt with during the critical stages of an infection. Such information is relevant not only for animal species, but also for humans, as currently, there is some active debate about the effects of dietary energy content on pathogen-induced anorexia and its consequences on the outcomes of infection in man (Wang et al., 2016). We followed the methodology of Sakkas et al. (2018) to separate the effects on feed intake during the different stages of infection: prepatent, acute, and recovery. This was to enable us to dissect the effects of diet composition on anorexia, and overcome some of the confusion that arises from the fact that the effects of infection on bird feed intake are often grouped together over variable periods of time (Lehman et al., 2009; Cloft et al., 2019a; Hilliar et al., 2020; Teng et al., 2021b).

Kyriazakis (2010, 2014) made some predictions about how diet composition may affect the characteristics of pathogen-induced anorexia, based on the assumption that anorexia is the outcome of the host attempt to regulate nutrient resource intake during the course of the infection. On the other hand, Sandberg et al. (2006), suggested that the extent of pathogen-induced anorexia is independent of host diet and only a function of the pathogen attributes, such as the infective dose, the kind of the pathogen etc. Their suggestion was based on the evidence that the interaction between host immune response and pathogen results in a cascade of physiological changes (e.g., cytokine response), which should result to the same degree of anorexia for a variety of pathogen attributes (Langhans, 2000). In other words, Sandberg et al. (2006) viewed anorexia as an unavoidable consequence of infection. The results of our experiments provide the first, to our knowledge, empirical evidence that anorexia is not the latter, but it can be manipulated by diet composition. To certain extents our findings are consistent with the expectations of Kyriazakis (2010) who suggested that there would be no advantage for the infected host to reduce further its ADFI when it is given access to diets of low nutrient resource content.

### The Relationship Between Anorexia and Dietary Energy Content at Constant Energy to Protein Ratio

In our first experiment we investigated the effect of diet composition on ADFI of broiler chickens infected with the coccidian parasite, *E. maxima,* when a high-quality diet was diluted by an inert material (lignocellulose, Arbocel). The dilution decreased simultaneously the energy can CP concentration of the diets. We expected that as the inclusion of Arbocel increased, the energy and CP bird intakes would decrease, due the limitations imposed on the ADFI by the increase in the bulk content of the diets (Nascimento et al., 2020). This was indeed the case for the uninfected birds throughout the experiments and for the infected birds during the prepatent and recovery stages of the infection. Both Taylor et al. (2021) and Nascimento et al. (2020) have suggested that the water holding capacity (**WHC**) of the diet might be the property of a diet that limits the voluntary ADFI of the birds. As indicated previously, Arbocel is a material with a high WHC and this was the reason it was chosen for the purposes of the experiment. It is possible that other fiber properties might be responsible for this limitation of ADFI through their effects on passage rate or gut capacity (González-Alvarado et al., 2010; Woyengo et al., 2017), but this was not among the objectives of our experiment.

The above effects of diet composition on ADFI were very different on infected birds during the acute stage of the infection. Although *Eimeria* infection resulted in the expected decrease in ADFI, in other words in pathogen-induced anorexia, diet composition had a significant effect on its extent during this stage only. As diet composition was diluted, ADFI actually increased rather than decreased. As a consequence of this, daily energy and CP intake of infected birds remained constant across diets. We interpret this interaction between diet and anorexia as an attempt by the hosts to regulate their nutrient resource intake during infection. Thus, different mechanisms appeared to regulate ADFI between uninfected and infected birds: bulkiness in the former and nutrient resource intake in the latter case.

A consequence of these effects on ADFI was that the outcomes of the infection were unaffected by diet composition. There were no significant differences in the ADG, numbers of excreted oocysts and damage to the gastrointestinal tract between the infected birds on the different diets. ADG of birds on the highest level of dilution (**HF**) during the acute phase of the infection was not significantly different from the best performance of infected birds achieved on the LF diet during the same stage of infection. This was in contrast with the performance of the uninfected birds during the acute stage of the infection, which reflected their ADFI: as dilution of the diet was increased, bird ADG gain was decreased. In fact, this was also the pattern in the ADG for both infected and uninfected birds during the prepatent and the recovery stages of the infection. We appreciate that inclusion of high levels of Arbocel in the diets could have resulted in reductions in the digestibility of nutrient resources (Röhe et al., 2020). As far as we are aware, there have been no investigations as to how the inclusion of lignocellulose affects nutrient digestibility in the presence of infection. However, in the presence of *Eimeria* challenge, Adedokun and Adeola (2016) have shown a reduction in the digestibility of both energy and nutrients in broilers infected with a live coccidian vaccine when offered a homogenous diet. Moreover, fiber inclusion may also affect endogenous losses in infected birds. Adedokun et al. (2012) observed a reduction in endogenous losses of *Eimeria* challenged broilers given a diet with 7.5% fiber compared to those given a diet with 2.5% fiber, whereas in nonchallenged birds there was no difference in endogenous losses between diets. If the birds in Experiment 1 responded similarly to those in Adedokun et al. (2012) then an increase in endogenous losses, and consequently a reduction in apparent digestibility, may account for the similarities in ADG of the infected LF and HF birds during the acute stage of infection.

Kyriazakis (2010) and Hilliar et al., (2020) have suggested that diet composition can affect the ability of animals to control their parasites through the functions of their immune response. *Eimeria* replication within the host enterocytes is regulated by the actions of the immune response (Rose, 1987), and consistent with other experiments on coccidian infections, infected birds in our experiment began to decrease the number of oocysts excreted by 7 d postinfection (Conway et al., 1999; Allen and Fetterer, 2002; Al-Badri and Barta, 2012). Given the same daily energy and CP intakes of the infected birds during the acute stages of the infection, it is then unsurprising that the development of oocyst excretion during the course of the infection was identical between birds on the different dietary treatments (Figure 2A).

Damage of the intestinal cells is a consistent outcome of *Eimeria* infection and mainly responsible for the consequences of the infection on the host (Yun et al., 2000; Kim et al., 2013). In the case of infection with *E. maxima*, the parasite typically penetrates the epithelial cells in the middle portion of the small intestine (Allen, 1997). However, there is evidence showing that the damage caused by *E. maxima* to the small intestine, in terms of VCR, can be extended from the duodenum to the ileum (Wils-Plotz et al., 2013; Sakkas et al., 2018). Infection had the expected effect on VCR, but diet composition during the acute stage of the infection (measurement taken on d6pi) had no effect on this infection outcome. This is consistent with the relationship between diet composition, extent of anorexia and the other infection outcomes, and can be seen as a strategy to maintain host resistance and tolerance to *Eimeria* infection. During the acute stage of infection (d6pi), the infected birds increased the relative length of the sections of the small intestine as diet dilution increased, whereas there was no difference between the uninfected birds for this measurement, an observation also seen in Bogusławska-Tryk et al. (2020). This interaction was only observed at d6pi, which could be interpreted as an attempt of the infected birds given the diluted diets to increase the absorptive area of the gastrointestinal tract during the peak of infection (Banfield et al., 2002).

### The Relationship Between Anorexia and Dietary CP Content at Constant Dietary Energy Content

Because of the manner the diets of Experiment 1 were formulated, that is, a constant nutrient to energy ratio, we cannot conclude whether birds were trying to regulate energy or nutrient, such as protein, intake during infection. This was tested in Experiment 2, where birds were offered diets of different CP contents, while their energy content remained constant; this was achieved by essentially exchanging corn gluten with corn starch, while maintaining the ratio of all AAs to both energy and lysine constant. Powdered cellulose (Solka-floc) was included in the diets as ‘balancer’ to facilitate the exchange of corn starch and corn gluten meal to achieve the diet formulations that enabled to address the experimental objectives. The inclusion levels of Solka-floc were very similar, ranging from 8.43 to 9.26%, across diets. Solka-floc is an inert ingredient with lower bulk properties (e.g., WHC) than Arbocel lignocellulose, used in Experiment 1 ( Boulos et al., 2000). It was therefore expected that Solka-floc would not limit the intakes of the birds through its bulkiness (Nascimento et al., 2020; Taylor et al., 2021), but the observed response would reflect the effect of dietary CP content on ADFI.

The outcomes of Experiment 2 in relation the effects of diet composition on pathogen-induced anorexia are not as clear cut as those of Experiment 1. For uninfected, control birds ADFI increased as the CP of the diet decreased during all stages of the experiment considered. However, the increase in ADFI was not sufficient to compensate for the decrease in the CP content, while at the same time being accompanied by an increase in daily energy intake. Whittemore et al. (2001) have suggested that animals attempt to eat for the first limiting resource in their diet. This suggestion is widely accepted for how animals respond to the dilution of dietary energy content, but it is less well accepted for their response to the dietary dilution of other nutrient resources (Misiura et al., 2020). The attempt of the uninfected birds to consume excess energy to meet their “targeted” daily CP intake can be seen within the context of the suggestion of Whittemore et al. (2001). It has been suggested that the lack of complete compensation in CP intake is because animals are limited in dealing with the excess energy intake that accompanies this response, due to their inability to dissipate it as heat in the environment (Kyriazakis et al., 1999).

Infected birds responded similarly to uninfected birds to the dilution of the CP content of their diet during all stages of the infection. However, there was a difference between infected and uninfected birds in their daily CP intake during the acute stage of the infection. CP intake was reduced most (2.6 g/d) in birds on the highest CP level (24%), whereas for all the other CP levels the reduction was similar at ∼1 g/d. Secondly, the CP intake of the infected birds on the 20% diet was similar to the CP intake of the uninfected birds on the same diet and similar to the CP intake of the infected birds on the 24% diet. We are tempted to interpret these responses as an attempt of the infected birds to regulate a certain level of daily CP intake during the acute stage of the infection. The fact that infected birds were not able to achieve such a CP “target” on the 16 and 12% diets could also be the consequence of bird inability to dissipate the accompanying excess energy intake, as heat into their environment. For smaller sized birds, such as the infected birds this effect would be even more acute and may be accentuated by pyrexia (Heinrich, 1977).

Unlike Experiment 1, oocyst excretion increased as the dietary CP content decreased; the increase was 7 times that of the 20% in the 12% CP dietary treatment. This effect could be seen as the outcome of the decreased CP intake and is consistent with the suggestions that the immune response is compromised by lower protein intake in several species infected by macro or macroparasites (Korver, 2012; Calder, 2013; Clough et al., 2016). Several immune mediators activated during primary *Eimeria* infections (cytokines, the acute phase protein response, lymphocyte proliferation, goblet cells, etc.) are proteinaceous in nature and as such they are expected to rely on protein intake (Teng et al., 2021a).

On the other hand, studies have shown that nutrient (e.g., AA) transporters are downregulated during the peak of *Eimeria* infection (Paris and Wong, 2013; Miska and Fetterer, 2018; Su et al., 2018). Paris and Wong (2013) suggested that this was to reduce the abundance of the primary energy source of the epithelial cells, leading to cell death and preventing parasite replication. Teng et al. (2021b) assessed the gene expression of nutrient transporters in broilers inoculated with graded doses of *E. maxima* oocysts. The results showed that the effects of infection on gene expression were not linear, yet the effects on growth performance were indeed linear, suggesting that the downregulation of nutrient transporters are not associated with the anorexia observed during *Eimeria* infection.

However, consistent with Experiment 1, ADG and villi length to crypt of infected birds were not affected significantly by dietary treatment. ADG was similar between birds on the 24, 20, and 16% diets during the acute stage of the infection. The fact that there were no differences in the damage (i.e., VCR) caused by the coccidian between the different CP diets, further suggests that birds minimized the consequence of the infection. This is consistent with Coltherd et al. (2009), who suggested that such (maintenance) functions are prioritized during infection. Maintenance of tissues associated with survival, such as the integrity of the gastrointestinal tract, ensures survival in the longer term (Garcia et al., 2018).

There is now consistent evidence that (healthy) animals and humans are targeting a specific protein intake when they are given a choice between different diets (Kyriazakis et al., 1999; Raubenheimer and Simpson, 2019). The mechanisms by how this regulation is achieved are currently being elucidated (Hill et al., 2020). Our experiments not only extend these suggestions to infected animals, but based on the reduction in CP intake of the birds fed the 24% CP diet they also suggest that the birds also avoided excess protein intake. This is in contrast to the results of Hilliar et al., (2020), who observed greater performance from d 7 to 35 pi in broilers given high CP diets during an *Eimeria* and necrotic enteritis (NE) challenge. Similarly, Lehman et al. (2009) showed that performance was improved in broilers given a high CP diet from d 1 to 42 pi. However, as previously mentioned, the contrasting conclusions on feeding high CP diets during infection are likely due to the time periods over which the data are considered. Evidence is growing that a certain level of protein intake during infection with *E. maxima* is beneficial both in terms of resilience and tolerance (Rochell et al., 2016; Yazdanabadi et al., 2020; Teng et al., 2021a). More specifically, AA profile is largely responsible for the effects on broiler growth and their responses to infection (Bortoluzzi et al., 2018). For example, Rochell et al. (2016) infected broilers with *E. acervulina* and offered the birds a series of low CP diets which were deficient in one of 8 AAs. The results highlighted the importance of AA profile rather than CP content; during the postinfection period, BW gain was significantly reduced in the low CP AA deficient treatments compared to the control, low CP treatment. Similar results were observed by Cloft et al. (2019b), where BW gain was improved in broilers given diets with a high AA density compared to low AA density from d 7 to 41 pi.

However, the question remains; why is an excess of protein intake, as in the case of the 24% CP diet, detrimental? There is some old evidence (Sharma et al., 1973) suggesting that high levels of CP increase the severity of coccidiosis in chickens, in terms of mortality and oocyst excretion. This has been ascribed to the higher levels of trypsin secretion caused by an increased dietary protein level, considered responsible for excystation of a larger proportion of infective oocysts (Britton et al., 1964). However, as this effect appears to be observed in other infections (e.g., with *Salmonella gallinarum* (Hill and Garren, 1961; Boyd and Edwards, 1963) and Newcastle (Boyd and Edwards, 1963) and Marek's disease viruses (Proudfoot and Aitken, 1969), there seems to be a generalised advantage to avoid excess of protein intake during the acute stages of the infection). Kyriazakis and Emmans (1991) suggested that in healthy animals at least, such an avoidance of excess protein intake was associated with the overloading of the metabolic processes in dealing with it. However, from an infected animal perspective, this could also be interpreted as the host reducing the risk of a secondary infection. Diseases such as coccidiosis can be predisposing factors for more severe infections, such as NE which is caused by the pathogenic bacterium *Clostridium perfringens* (Prescott et al., 2016). Hilliar et al., (2020) suggested that feeding low CP diets could theoretically negate the effects of NE infection as there are lower amounts of undigested protein to support the proliferation of protein fermenting pathogenic bacteria, such as *C. perfringens* (Drew et al., 2004; Wu et al., 2014). Such bacteria challenge gastrointestinal gut health and may lead to local and systemic inflammation. Therefore, it seems logical for the host to limit protein intake in order to reduce the risk of succumbing to further infections, though this requires further investigation.

## CONCLUSION

There has been a lack of understanding about how diet composition affects pathogen-induced anorexia and consequentially the outcomes of infections in both animals and humans. Here we show that such anorexia is sensitive to diet composition and that it may altogether be absent in diets of low nutrient density. During infection with *Eimeria*, birds targeted a protein intake which appeared to be beneficial for the outcome of the infection, while at the same time they avoided excess protein. We suggest that excess protein intake may be associated with detrimental consequences in the ability of the host to cope with pathogens, but this would need to be tested.

Although we have shown the consequences of the regulation of nutrient resource intake through anorexia on the outcomes of *Eimeria* infection, the question about the mechanisms by which these are achieved remains. We suggest that this is a research area where future effort could be fruitfully directed to.

## Appendix Supplementary materials

Image, application 1

## Footnotes

Supplementary material associated with this article can be found, in the online version, at doi:10.1016/j.psj.2021.101535.
