# Supplementary material for: Starving for nutrients: anorexia during infection with parasites in broilers is affected by diet composition
Source: Poult Sci. 2021 Oct 13;101(1):101535. doi: 10.1016/j.psj.2021.101535 (PMC8605289; doi:10.1016/j.psj.2021.101535)
Supplement: Supplementary file 1 [file mmc1.docx]

**Figure S1.** Pen average body weight (g) of infected (I, dotted lines) and the corresponding uninfected control (C, solid lines) birds at the start (d0pi) and end (d15pi) of infection with *Eimeria maxima* oocysts in Experiment 1. Birds were offered diets diluted with 0% (NF), 5% (LF), 10% (MF), or 15% (HF) Arbocel lignocellulose; diet NF contained 3105 kcal/ kg ME and 19.4% CP. As a subset of birds were removed for intestinal tissue measurements the means are based on different bird numbers: there were 6 birds per pen until d6pi, 5 birds per pen until d12pi and 4 birds per pen until d15pi.

**Figure S2.** Pen average body weight (g) of infected (I, dotted lines) and the corresponding uninfected control (C, solid lines) birds at the start (d0pi) and end (d15pi) of infection with *Eimeria maxima* oocysts in Experiment 2. Birds were offered diets with 24%, 20%, 16%, or 12% CP, which were isonitrogenous (3105 kcal/ kg ME). As a subset of birds were removed for intestinal tissue measurements the means are based on different bird numbers: there were 6 birds per pen until d6pi, 5 birds per pen until d12pi and 4 birds per pen until d15pi.

**Table S1.** Small intestine segment relative lengths of infected and the corresponding uninfected control birds during infection with *Eimeria maxima* oocysts in Experiment 1**.** The lengths of the duodenum (DL), jejunum (JL), and ileum (IL) (cm) were scaled per unit of kg body weight (BW) at the point of euthanasia, at d6 and d12 post-infection. Birds were offered diets diluted with 0% (NF), 5% (LF), 10% (MF), or 15% (HF) Arbocel lignocellulose, diet NF contained 3105 kcal/ kg ME and 19.4% CP.

|  |  | D6pi | | | D12pi | | | |  |
| --- | --- | --- | --- | --- | --- | --- | --- | --- | --- |
|  |  | DL (cm/ kg BW) | JL (cm/ kg BW) | IL * (cm/ kg BW) | DL (cm/ kg BW) | JL (cm/ kg BW) | IL (cm/ kg BW) | |  |
| Diet |  |  |  |  |  |  |  | |  |
|  | NF | 31.6^y^ | 81.5^x^ | 4.41^y^ | 26.0^yz^ | 64.9 | 64.7 | |  |
|  | LF | 34.6^yz^ | 91.3^xy^ | 4.47^y^ | 23.0^y^ | 63.9 | 69.2 | |  |
|  | MF | 38.9^z^ | 101^yz^ | 4.68^z^ | 27.1^z^ | 69.2 | 70.5 | |  |
|  | HF | 39.0^z^ | 111^z^ | 4.70^z^ | 28.1^z^ | 71.3 | 75.3 | |  |
|  | SEM | 1.87 | 2.96 | 0.030 | 0.94 | 3.27 | 2.82 | |  |
| Infection |  |  |  |  |  |  |  | |  |
|  | Control | 33.1 | 84.2 | 4.46 | 23.9 | 61.3 | 64.2 | |  |
|  | Infected | 38.9 | 108 | 4.67 | 28.1 | 73.4 | 75.6 | |  |
|  | SEM | 1.32 | 2.32 | 0.03 | 0.67 | 2.31 | 2.00 | |  |
| Diet × Infection |  |  |  |  |  |  |  | |  |
| Uninfected | NF | 31.2^a^ | 83.7^a^ | 4.39^a^ | 22.8 | 56.8 | 54.4 | |  |
|  | LF | 32.6^ab^ | 78.1^a^ | 4.46^a^ | 21.3 | 57.7 | 64.7 | |  |
|  | MF | 35.1^abc^ | 84.9^a^ | 4.44^a^ | 25.9 | 68.4 | 69.3 | |  |
|  | HF | 33.6^abc^ | 90.0^a^ | 4.57^a^ | 25.7 | 62.2 | 68.5 | |  |
|  |  |  |  |  |  |  |  | |  |
| Infected | NF | 32.1^a^ | 79.3^a^ | 4.44^a^ | 29.1 | 73.0 | 74.9 | |  |
|  | LF | 45.3^c^ | 125^b^ | 4.90^b^ | 24.6 | 70.2 | 73.8 | |  |
|  | MF | 34.1^abc^ | 97.7^a^ | 4.51^a^ | 28.4 | 69.9 | 71.6 | |  |
|  | HF | 44.3^bc^ | 132^b^ | 4.84^b^ | 30.5 | 80.4 | 82.2 | |  |
|  | SEM | 2.64 | 4.37 | 6.15 | 1.33 | 4.63 | 3.99 | |  |
|  |  | *Probabilities* | | | | | |  |  |
| Diet |  | **0.019** | **<0.001** | **<0.001** | **0.003** | 0.343 | 0.081 | |  |
| Infection |  | **0.003** | **<0.001** | **<0.001** | **<0.001** | **0.001** | **<0.001** | |  |
| Diet × Infection |  | **0.028** | **<0.001** | **0.002** | 0.518 | 0.289 | 0.153 | |  |

^a-c, x-z^ Means within a column that do not share a common superscript are significantly different (*P* < 0.05).
* indicates data were log transformed for analysis.

**Table S2.** Small intestine segment relative lengths of infected and the corresponding uninfected control birds during infection with *Eimeria maxima* oocysts in Experiment 2**.** The lengths of the duodenum (DL), jejunum (JL), and ileum (IL) (cm) were scaled per unit of kg body weight (BW) at the point of euthanasia, at d6 and d12 post-infection. Birds were offered diets with 24%, 20%, 16%, or 12% CP, which were isonitrogenous (3105 kcal/ kg ME).

|  |  | D6pi | | | D12pi | | | |  |
| --- | --- | --- | --- | --- | --- | --- | --- | --- | --- |
|  |  | DL (cm/ kg BW) | JL (cm/ kg BW) | IL (cm/ kg BW) | DL (cm/ kg BW) | JL (cm/ kg BW) | IL (cm/ kg BW) | |  |
| Diet |  |  |  |  |  |  |  | |  |
|  | 24 | 34.1^y^ | 89.7^y^ | 78.6^x^ | 30.3^xy^ | 70.8^xy^ | 64.4^y^ | |  |
|  | 20 | 35.0^y^ | 91.6^y^ | 88.0^y^ | 27.9^x^ | 67.2^x^ | 60.5^y^ | |  |
|  | 16 | 36.7^y^ | 97.1^yz^ | 87.3^xy^ | 31.3^y^ | 75.7^y^ | 67.5^y^ | |  |
|  | 12 | 40.8^z^ | 108^z^ | 101^z^ | 36.3^z^ | 92.5^z^ | 86.0^z^ | |  |
|  | SEM | 1.38 | 3.49 | 2.39 | 0.81 | 1.67 | 2.18 | |  |
| Infection |  |  |  |  |  |  |  | |  |
|  | Control | 35.6 | 91.1 | 88.2 | 28.2 | 67.6 | 62.5 | |  |
|  | Infected | 37.8 | 102 | 89.5 | 34.7 | 85.5 | 76.7 | |  |
|  | SEM | 0.98 | 2.47 | 1.69 | 0.57 | 1.18 | 1.54 | |  |
| Diet × Infection |  |  |  |  |  |  |  | |  |
| Uninfected | 24 | 34.1 | 86.6 | 77.4 | 26.6 | 61.3 | 56.5 | |  |
|  | 20 | 33.6 | 83.0 | 87.5 | 26.3 | 60.9 | 57.6 | |  |
|  | 16 | 35.7 | 96.4 | 86.5 | 27.7 | 67.4 | 59.0 | |  |
|  | 12 | 38.9 | 98.5 | 101 | 32.3 | 80.8 | 76.8 | |  |
|  |  |  |  |  |  |  |  | |  |
| Infected | 24 | 34.2 | 92.8 | 79.8 | 34.1 | 80.3 | 72.4 | |  |
|  | 20 | 36.5 | 100 | 88.6 | 29.5 | 73.5 | 63.4 | |  |
|  | 16 | 37.7 | 97.9 | 88.1 | 35.0 | 84.1 | 76.0 | |  |
|  | 12 | 42.7 | 118 | 102 | 40.3 | 104 | 95.1 | |  |
|  | SEM | 1.95 | 4.93 | 3.38 | 1.14 | 2.36 | 3.08 | |  |
|  |  | *Probabilities* | | | | | |  |  |
| Diet |  | **0.007** | **0.003** | **<0.001** | **0.007** | **<0.001** | **<0.001** | |  |
| Infection |  | 0.119 | **0.003** | 0.567 | 0.119 | **<0.001** | **<0.001** | |  |
| Diet × Infection |  | 0.806 | 0.233 | 0.993 | 0.806 | 0.165 | 0.181 | |  |

^x-z^ Means within a column that do not share a common superscript are significantly different (*P* < 0.05).
